# Effects of PEF-Assisted Freeze-Drying on Protein Quality, Microstructure, and Digestibility in Chilean Abalone “Loco” (*Concholepas concholepas*) Mollusk

**DOI:** 10.3389/fnut.2022.810827

**Published:** 2022-03-17

**Authors:** Anais Palma-Acevedo, Mario Pérez-Won, Gipsy Tabilo-Munizaga, Jaime Ortiz-Viedma, Roberto Lemus-Mondaca

**Affiliations:** ^1^Departamento de Ingeniería en Alimentos, Facultad de Ciencias de la Salud y de los Alimentos, Universidad del Bío-Bío, Chillán, Chile; ^2^Departamento de Ciencia de los Alimentos y Tecnología Química, Facultad de Ciencias Químicas y Farmacéuticas, Universidad de Chile, Santiago, Chile

**Keywords:** Chilean abalone, freezing-drying, pulsed electric field, thermal behavior, structural quality

## Abstract

The purpose of this study was to apply different pulsed electric field (PEF) conditions as a pretreatment to the freeze-drying (FD) process of Chilean abalone and to assess its effects on protein quality, microstructure, and digestibility of the freeze-dried product. The treatments PEF (0.5, 1.0, and 2.0 kV cm^−1^) and cooking (CO) were applied at 100°C × 15 min. Then, their performances were subjected to a FD process. PEF + CO pretreated freeze-dried samples showed shorter process times than freeze-dried control samples without PEF + CO, where the treatment PEF at 2.0 kV cm^−1^ reached the shortest time. In addition, the abovementioned samples presented the best textural parameters but a low protein content. The thermal properties indicate a total denaturation of the proteins, where the amide I region presented greater mobility in the sample pretreated with an electric field of 2.0 kV cm^−1^. The assay for digestibility shows better hydrolysis for the 2.0 kV cm^−1^ PEF sample and has a higher Computer-Protein Efficiency Ratio (C-PER). Thereby, variations in thermal behavior and physicochemical parameters in comparison to combined PEF + CO pretreatments were observed. In addition, high protein quality and digestibility of pretreated freeze-dried Chilean abalones were maintained to the desired properties (texture and C-PER) and conditions (FD time).

**Graphical Abstract:**
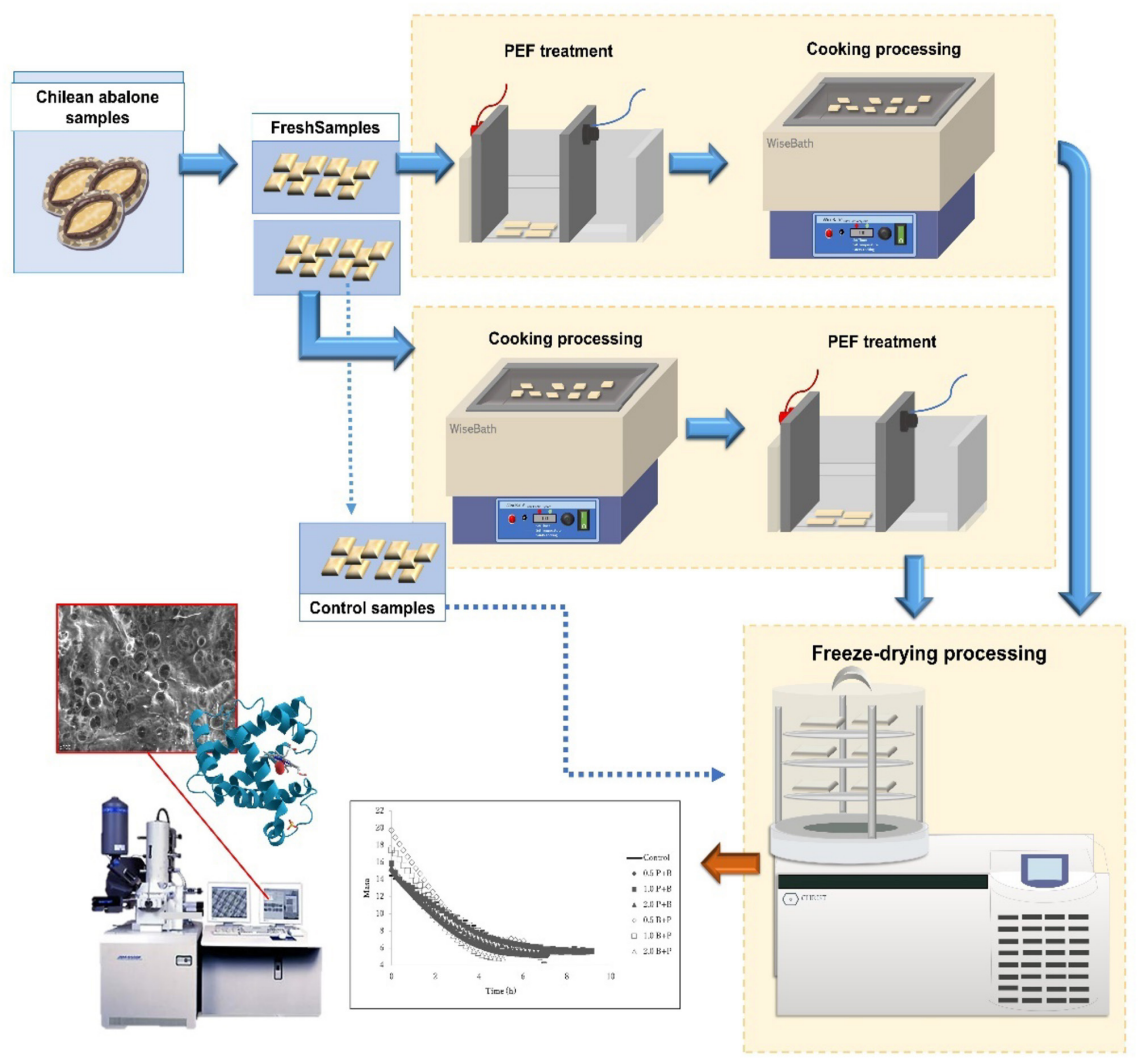
Schema of general Chilean abalone processing by PEF-assisted freeze-drying, and parts of the Chilean abalone used for physicochemical analysis are illustrated.

## Introduction

Chilean abalone (*Concholepas concholepas*) mollusks are colloquially known as “Loco,” which are considered exotic and expensive seafood. However, one of the main factors limiting seafood export is its short shelf-life (1). In both North America and Asian countries, Chilean abalone is enjoyed for its tenderness of properly cooked meat (2). The Chilean abalone, a mollusk very similar to other abalone species (e.g., *Haliotis rufescens* and *Haliotis discus* hannai), is endemic from the Chile and Peru coasts (3). Furthermore, processed Chilean abalone is a gourmet product of high commercial value in both Chile and international markets due to its main sensorial attributes of texture and flavor (4, 5). Raw mollusks have high hardness related to the availability of muscle proteins, with a high content of paramyosin bound to collagen has been found in this muscle along with actomyosin (6), which is the main myofibrillar protein attributed to changes in meat toughness (7).

Currently, pulsed electric field (PEF) technology used in food processing is considered an emerging technology that could replace traditional pasteurization processes (8). High-intensity PEF equipment consists of several devices, including a power supply, a capacitor bank, a switch, the treatment chamber, electric current, temperature sensors, and in some cases, an aseptic packaging system (9). PEF technology is also applied as a pretreatment in the food industry, and there are several studies about its application to alcoholic beverages, eggs, and dairy products (10). Accordingly, PEF technology applied to marine products would be favorable because of its short processing times and low energy consumption.

Likewise, the cooking (CO) process is also a widely used pretreatment, specifically, to minimize heat damage on food quality as well as reduce process drying times (11). Thereby, the CO process has been combined with different processing methods such as HHP, PEF, and ultrasound (3, 11). Particularly, using HHP before food drying significantly increased mass transfer during drying (12, 13), which reduced the exposure time of the food to oxygen. Applying CO and PEF as pretreatments could increase the drying rate by inducing changes in the protein structure and increasing the moisture diffusion rate during the freeze-drying (FD) process.

Freeze-drying is a drying technique in which the food product is initially frozen, and the water content is then converted directly from ice to vapor by very low-pressure sublimation (3). This methodology allows the structure of the food matrix to remain in an unaltered position to preserve color and flavor (14). Therefore, the use of FD by the food industry is normally restricted to high-value products, such as coffee, crisp fruits, and vegetables, ingredients for ready-to-eat foods, and some aromatic herbs (15).

Protein quality assessment can be measured by using different methods. Fourier transform IR (FTIR) spectroscopy and differential scanning calorimetry (DSC) are considered suitable analytical techniques to evaluate thermal and structural changes in proteins (16). DSC provides information at the physical and macroscopic level by indicating changes associated with gelatinization or denaturation phenomena, while FTIR spectroscopy gives more detailed structural information through the evaluation of the secondary structure of proteins (17).

Thermophysical properties in food science and technology are used to calculate loads and heat fluxes and to establish standards for critical points during a process (18, 19). In addition, these properties are required to estimate process times for refrigerating, freezing, heating, or drying foods (18). The thermophysical properties so far published have served to increase the database as well as to allow and test the mathematical models that depend on the composition, temperature, pressure, orientation of the fiber, etc. (20). As for the food microstructure, its evaluation depends on the processing method, the operating variables, and the microstructure of the food produced during the process (21).

Therefore, this work aimed to assess the influence of combined pretreatments, CO and PEF, on the FD process kinetics, protein thermal behavior, and microstructure of Chilean abalone “Loco” mollusk, as well as protein *in vitro* digestibility.

## Materials and Methods

### Raw Material

Chilean abalone samples (without shell) were obtained from a local market in Coquimbo city, Chile. The sample size was between 4 and 6 cm, and the average weight was 50.0 ± 0.5 g. The samples were transported in ice, and they were later cut into slices of 20 × 30 × 3 mm. Then, they were stored at 4.0 ± 0.2°C for no more than 48 h until further processing.

### Pretreatments and FD Process

The pulsed electric field process was applied with a semiconductor-based positive Marx modulator Epulsus-PM1–10 equipped with a batch treatment chamber (EPULSUS®, PMX-Y, Portugal). A parallel plate treatment chamber that consisted of stainless-steel electrodes with a 140 cm^2^ electrode area and a 10 cm gap was used. The PEF working conditions at room temperature were as follows: pulse width of 15 μs, frequency of 1 Hz, 50 pulses, 5 cm gap, and electric fields of 0.5, 1.0, and 2.0 kV cm^−1^ for each pretreatment, and then they were cooked at 100°C for 15 min in a ratio of 50 ml of distilled water for 1 g in a thermoregulated bath (WiseBath, Wisd, Germany), this first group was labeled as “kV cm^−1^ PEF + CO.” While a second group was subjected to the same pretreatments and conditions but inversely, that is, first CO and then PEF, which was labeled “CO + kV cm^−1^ PEF.” Each batch was 15 ± 0.5 g. From the abovementioned details, both samples from groups were frozen at −55°C and then lyophilized at 0.021 mbar (Christ Alpha, 3-4 LD basic, Denmark). A sample without pretreatments submitted to the FD process was considered a control sample. However, to have the effect of CO and PEF pretreatments of the Chilean abalone on FD, a cooked-only sample a 2.0 kV cm^−1^ treated only sample submitted for freeze-drying and included in the analyses were labeled as “Cooked” and “2.0 PEF,” respectively. All the experiments were performed three times.

### Moisture and Protein Content

Moisture content was determined by AOAC methodology No. 934.06 (22). Total protein content was determined by the AOAC method (2005), which is based on the adapted Kjeldahl method. Protein content was calculated using the following equation:


(1) $$\begin{array}{l} \%Prot=\frac{\left[H_{2}SO_{4}\right]\times \left(V_{f}-V_{i}\right)\times F}{w_{s}} \end{array}$$

where [H_2_SO_4_] is the concentration of the titled solution, *V*_*f*_ is the final volume, *V*_*i*_ is the initial volume, *F* is the factor of protein source (6.25), and *w*_*s*_ is sample weight.

### Rehydration Indexes

Freeze-dried samples were placed in distilled water at 100°C for 6 h, using a solid to liquid ratio of 1:50. The samples were then removed, drained for 30 s, and weighed. Rehydration capacity (RC) was calculated according to Equation (2). Water holding capacity (WHC) was determined by centrifuging the rehydrated samples at 3,500 × *g* for 15 min at 20°C in tubes fitted with a centrally placed plastic mesh, which allowed water to drain freely from the sample during centrifugation. WHC was calculated from Equation (3).


(2) $$\begin{array}{l} RC=\frac{\left(W_{r}\times X_{r}\right)-\left(W_{d}\times X_{d}\right)}{W_{d}\times \left(1-X_{d}\right)} \end{array}$$

(3) $$\begin{array}{l} WHC=\frac{\left(W_{r}\times X_{r}\right)-W_{dl}}{\left(W_{r}\times X_{r}\right)} \end{array}$$

where *W*_*r*_ is the weight of the sample after the rehydration process, *X*_*r*_ is the corresponding moisture content on a wet basis, *W*_*d*_ is the weight of the sample after the drying process, *X*_*d*_ is the corresponding moisture content on a wet matter, and *W*_*dl*_ is the weight of the drained liquid after centrifugation.

### Texture Profile Analysis

The texture profile analysis (TPA) was carried out using a texture analyzer (Model TA-TX PLUS, Texture Technologies, Scarsdale, NY, USA). The sample was compressed to 75% of its original thickness with a speed of 1 mm/s, with a 35-mm-diameter aluminum cylinder. Texture analysis was automatically performed using the Texture Expert software v.2.63 (Stable Micro Systems Ltd., Godalming, UK), and the following parameters were recorded: hardness, cohesiveness, springiness, and chewiness (23). A total of 10 measurements were performed for each analysis.

### DSC Thermal Behavior

Enthalpy (ΔH), fusion temperature (Tm), specific heat (Cp), and transition glass temperature (Tg) were determined using DSC (DSC 1, Star System, Shimadzu Corporation, Kyoto, Japan) equipped with a low-temperature cooling unit (LTC-50, Shimadzu Corporation, Kyoto, Japan). The samples were weighed and placed in hermetically sealed 100-μl aluminum pans (19 mg approx.) and then loaded onto the DSC support at room temperature, using an empty pan as a reference. Samples were then cooled at 25°C/min for 5 min and warmed up at a rate of 30–200°C under an N_2_ inert gas atmosphere (50 ml/min). Tg is reported as the midpoint of the step. ΔH and ΔCp were estimated for each sample by measuring the area under the DSC transition curve (Stareware, v.10.01 Mettler Toledo, Schwerzenbach, Switzerland).

### FTIR Spectroscopy

IR spectra measurements of 1 mg for each sample at room temperature were obtained using the FTIR equipment (Shimadzu Corporation Pte. Ltd., Kyoto, Japan) by setting 128 scans at 4.0 cm^−1^ spectral resolution. Temperature-dependent spectra were obtained using a spectra variable temperature IR cell controlled by a Red Lions digital temperature controller; 64 scans were co-added. The IR spectra ranged from 1,700 to 1,600 cm^−1^ (amide I region). The resulting spectra were mathematically treated with IRsolution software v.1.10 (Creon Lab Control AG, Shimadzu Corporation Pte. Ltd., Kyoto, Japan), to remove possible noises, the spectra were smoothened and derived from a 12-point Savitsky–Golay function.

### Determination of *in vitro* Gastrointestinal Digestibility

*In vitro* gastrointestinal digestibility was carried out according to Levi and Lesmes (24). Two bioreactors (MiniBio, Applikon Biotechnology, Netherlands) were serially connected through a silicon tube (115 cm in length, Medent, Israel cat. 054-010030) to simulate gastric and duodenal phase digestion of a healthy adult, which were computer-controlled through a specialized program “my-Control” software version 1.0X (Applikon, Netherlands) as well as all processes, including temperature, pH gradient, time, and pump. Gastric bioreactor conditions were set at a temperature of 37°C, with an initial volume of 90 ml simulated gastric fluids (SGF), plus 1.7 g of rehydrated, manually minced Chilean abalone in 10 ml of water, 200 RPM as a stirrer rate according to Levi and Lesmes (24), and 20 s pulses, 1–2 times per min in average. SGF consisted of a solution of KCl 0.63 M, KH_2_PO_4_ 0.5 M, NaHCO_3_ 1 M, NaCl 2 M, MgCl_2_ × 6H_2_O 0.15 M, NH_4_Cl 0.51 M, Urea 0.38 M, CaCl_2_ × 2H_2_O 4 M. Subsequently, an Intestinal bioreactor was set and filled with an initial volume of 10 ml of simulated duodenal fluids (SDF) and consisted of a solution of KCl 0.63 M, KH_2_PO_4_ 0.5 M, NaHCO_3_ 1 M, NaCl 2 M, MgCl_2_ × 6H_2_O 0.15 M, Urea 0.38 M, CaCl_2_ × 2H_2_O 4 M, HCl 1M, NaOH 1M. For pH adjustment, stock solutions of HCl 32% and NaOH 5 M were necessary. All solutions were prepared using MiliQ water. Time processing was set at 2 h. Samples were aseptically collected from each bioreactor through a designated tubing system located in the vessel head plate BioXpert V2 v.2.93 (Applikon, Netherland) in the times of 0, 60, 90, and 120 min. All experiments were performed three times. The assay was applied to the chosen samples according to shorter FD kinetic times, the analyzed samples were FD without pretreatments (control) and FD with 2.0 CO + PEF and 2.0 PEF pretreated samples.

### Determination of Degree of Hydrolysis

The degree of hydrolysis (DH) was measured by using the σ-Phthaldialdehyde (OPA) method according to Nielsen et al. (25) under intervals of 0, 60, 90, and 120 min. OPA reagent was prepared as follows: 160 mg of OPA were dissolved in 4 ml ethanol and then were added to a previously prepared solution containing 7.62 g borax (Na_2_[B4O5(OH)4]·10H_2_O), and 200 mg of SDS (NaC1_2_H_25_SO_4_) dissolved on 150 ml deionized water. The already mixed solutions, 176 mg dithiothreitol (DTT) 99% was added and was placed and filled into a 200-ml volumetric flask. The final reagent was stored in an amber bottle and used on the same day. A calibration curve was obtained using L-serine solution in the range of 50–200 mg/ml (25). The assay was carried out by pipetting 0.5 ml of the sample in an Eppendorf tube of 1 ml and centrifuged for 20 min, 14,000 *g*, room temperature. A total of 200 μl of supernatant were mixed to 1.5 ml of the OPA reagent for further absorbance measurement at 340 nm after 3 min of incubation (Thermo Scientific, Genesys 10S UV-Vis, Madrid).


(4) $$\begin{array}{l} DH=\frac{h}{h_{tot}}\times 100\% \end{array}$$

(5) $$\begin{array}{l} h=\frac{Srine\;NH_{2}-\beta }{\alpha } \end{array}$$

where serine NH_2_ = meqv serine NH_2_/g protein, α and β are published by Nielsen et al. (25) for specific raw materials.

### Digested Protein Efficiency

The calculated protein efficiency ratio (C-PER) was determined using the procedure described by Phimphialai et al. (26) and Satterlee et al. (27) and the data were obtained from Sindayikengera and Xia (28) and Chávez-Mardones et al. (29) using Equation (6). Each essential amino acid (EEA) was described as a standard percentage from the FAO/WHO:


(6) $$\begin{array}{l} \%EAA_{FA0}=\frac{\left[\frac{gEAA^{\;}}{100g\;protein}\right]\times \left[in\;vitro\;protein\;digestibility\right]}{FAO/WHO\;std.\;for\;that\;EAA} \end{array}$$

Subsequently, the %EAA_FAO_ was adjusted as follows:

If all %EAA_FAO_ are ≤100%, continue in the normal way with computation using Equations (6) and (7), however, if %EAA_FAO_ ≥ 100%, reduce to 100% and continue with the same equations (26):


(7) $$\begin{array}{l} X\;=\sum \left(\frac{1}{\left[\%EAA_{FAO}]\;\times \;[associated\;weight\right]}\right) \end{array}$$

(8) $$\begin{array}{l} Y\;=\sum \left(associated\;weight\right) \end{array}$$

### Microstructural Analysis

Pretreated and non-pretreated freeze-dried samples were placed on a watch glass and then were cut to a thin, uniform cross-section with scalpel and forceps. The sectioned sample was deposited on a piece of double-sided carbon fiber tape, which allows a better quality in the micrograph. Finally, the samples were deposited one by one on the sample holder of the SEM equipment (Hitachi, SU3500, Japan) and introduced into its vacuum chamber. The optimal conditions necessary for capturing micrographs were calibrated with the magnification of 65X, 200X, and 500X for the lengths of 500, 200, and 50 μm, respectively.

### Statistical Evaluation

All data were expressed as mean values ± SD. Statistical analysis of data was carried out using Statgraphics Plus® software v.5.1, applying an ANOVA to estimate significant differences applying the Tukey test for a confidence level of 95% (*p* < 0.05), and a multiple range test (MRT) was also used to determine possible homogeneous groups between treatments.

## Results and Discussion

### FD Kinetics

**Figure 1:**
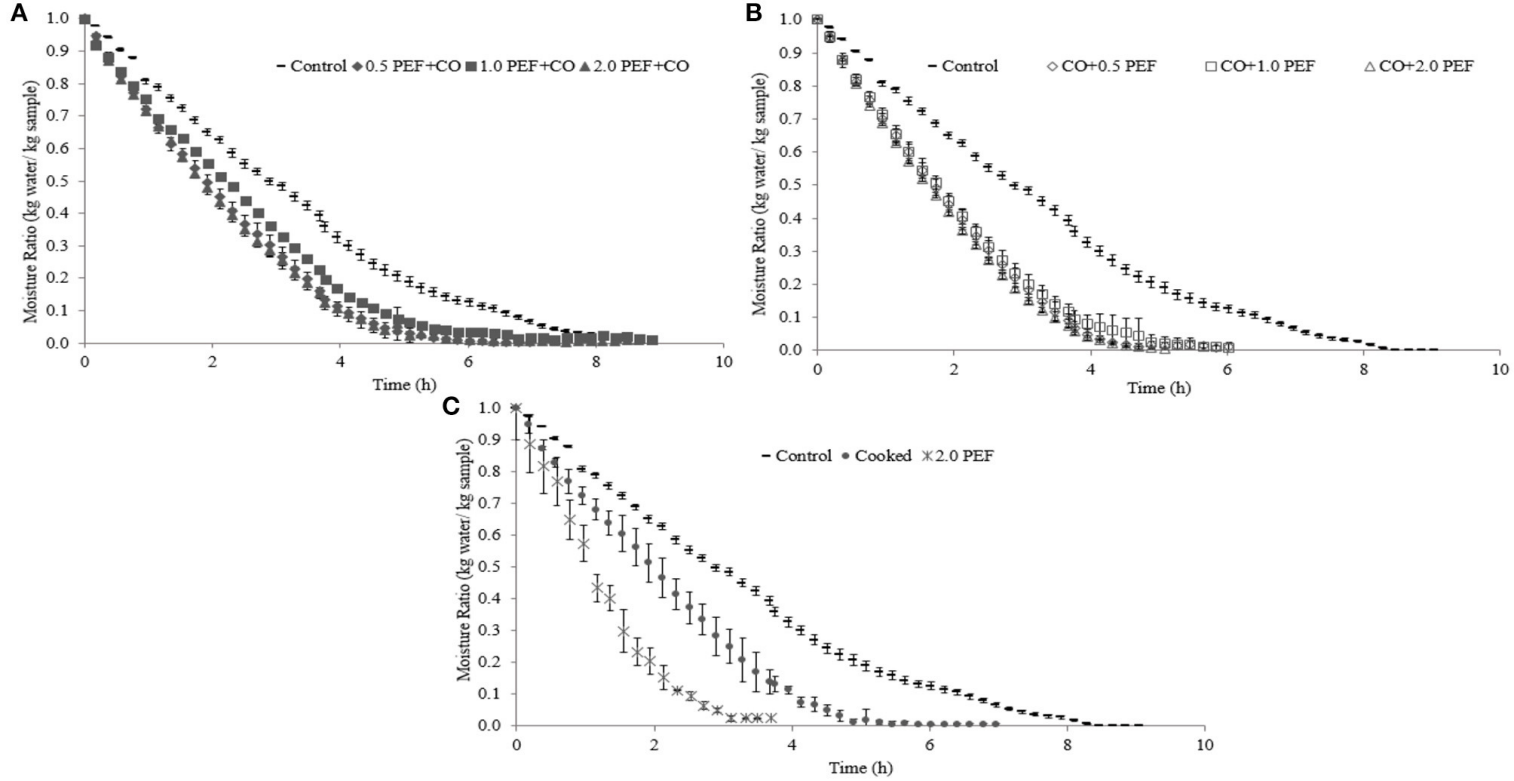
Freeze-drying (FD) kinetics of samples under different pretreatment conditions: **(A)** Pulsed electric field (PEF) + cooking (CO) pretreated samples compared to control sample, **(B)** CO + PEF pretreated samples compared to control sample, and **(C)** 2.0 PE or CO pretreated samples compared to control sample.

Figure 1 shows the FD kinetics of PEF and CO pretreated Chilean abalone samples. The freeze-dried control sample without pretreatments took around 9 h to reach the equilibrium moisture ratio (MR) as shown in Figure 1. However, the FD time taken to get the MR value under the different PEF and CO conditions varied between 3 and 8 h. In that way, FD times under pretreatments represented were shorter at CO, 0.5 PEF+CO, 1.0 PEF+CO, 2.0 PEF+CO, with times of 6.57, 6.97, 8.85, and 8.67 h, respectively. However, the process conditions for 2.0 PEF, 0.5 CO + PEF, 1.0 CO + PEF, 2.0 CO + PEF showed times much shorter than 6.02, 6.25, 5.08 to 3.69 h, respectively. In these cases, the effect of PEF was significant when applied after CO, always taking into account the moment in which the technology is applied.

Other authors also evaluated different pretreatment technologies for diverse drying methods and always reached reduced process times. Aksoy et al. (38) evaluated the effect of ultrasound-assisted vacuum drying (USV) and vacuum drying (VD) on minced meat, and the results showed a drying time of 1.5 h and a longer drying of 3.3 h using the USV technique and the VD method, respectively. Moreover, Elmas et al. (30) assessed the influence of pre-drying methods on fresh turkey breast meat. The drying techniques used were microwave (MW), hot air drying (HD), and FD prior to puff drying. In this case, the HD and FD pre-dried samples had higher and lower moisture content, respectively (12.3% HD and 2.31% FD), with final time puff-drying of 2.8 h for HD and 3.92 h for FD. From this, this study reported drying times to be longer than the earlier studies due to increased surface exposure from the minced meat. In essence, the porosity of the food sample increases with less shrinkage, which, in turn, favors the rehydration ratio of the FD samples (31).

Although PEF processing has been used as a pretreatment in the dried food industry for diverse vegetables (20, 32–35). Nevertheless, there are few previous works on the effect of PEF as a pretreatment for the drying process in matrices of animal origin; however, an investigation carried out by Astráin-Redín et al. (20) reported a reduction in the curing time of Spanish sausage between 19 and 33% when applying PEF as a pretreatment.

### Physicochemical Properties

As shown in Table 1, moisture content was decreased by the application of pretreatments as all the pretreated samples presented statistically significant differences between them (*p* < 0.05), and values lower than control sample moisture content. The final moisture values (Table 1) are lower than those obtained by Reyes et al. (5), who lyophilized Chilean abalones without pretreatments, so the difference could be due to the application of both PEF and CO pretreatments. On the other hand, the final moisture content (1.5% approx.) of 2.0 PEF + CO treated sample compared to CO + 2.0 PEF treated sample indicated that both pretreatment application order of and PEF pretreatment magnitude significantly affected the final moisture content. Presumably, the pretreatments could lead to a decrease of WHC of muscle protein, which contributed to the uptake of water in the extra myofibrillar space of muscles (39). Additionally, water loss may also be due to myofibrillar denaturation (36).

**Table 1:** Final moisture content, water retention capacity, and protein content of pretreated and non-pretreated freeze-dried samples.

**Treatment**	**Moisture (% d.b.)**	WHC (^*^)	RR (^**^)	**Protein (% d.b.)**
Control	5.9 ± 0.1^a^	98.7 ± 0.2^a^	1.9 ± 0.1^a^	60.2 ± 0.3^bc^
0.5 PEF+CO	3.4 ± 0.2^d^	98.7 ± 0.1^a^	1.6 ± 0.4^a^	61.6 ± 0.5^bc^
1.0 PEF+CO	4.2 ± 0.2^c^	97.8 ± 0.5^a^	1.6 ± 0.1^a^	74.0 ± 0.2^ab^
2.0 PEF+CO	1.5 ± 0.0^e^	94.2 ± 3.0^b^	1.7 ± 0.0^a^	83.7 ± 1.4^a^
CO+ 0.5 PEF	3.2 ± 0.3^d^	97.1 ± 0.4^a^	1.7 ± 0.3^a^	66.6 ± 1.9^bc^
CO+1.0 PEF	4.3 ± 0.4^c^	98.8 ± 0.3^a^	1.7 ± 0.1^a^	68.7 ± 0.7^bc^
CO+2.0 PEF	1.9 ± 0.4^e^	96.2 ± 0.6^a^	1.8 ± 0.1^a^	84.3 ± 0.7^a^
Cooked	4.2± 0.2^c^	98.7± 0.3^a^	1.1± 0.0^b^	67.7 ± 0.3^bc^
2.0 PEF	5.0± 0.4^ab^	95.6 ± 0.1^b^	1.1 ± 0.2^b^	65.9 ± 0.6^bc^

*Lowercase letters indicate the statistical significance (p < 0.05) among all the treatments compared with each other (column)*.

* *Water holding capacity (WHC): g retained water/100 g water*,

** *RR: g absorbed water/g d.m*.

The WHC value of 2.0 PEF + CO pretreatment (Table 1) showed a significant difference from the other pretreatments, this value (94.2 g retained water/100 g water) is related to its moisture content before being obtained, the lowest one, which could indicate that PEF application at 2.0 kV cm^−1^ induced an opening pore that was kept open by the subsequent CO application. Both ANOVA analysis and MRT were performed on WHC values for PEF pretreated samples (i.e., 0.5 PEF + CO with CO + 0.5 PEF), and no significant differences were found (data not shown) when comparing this treatment with each other, including the control. The detected non-statistical differences could be due to the degree of denaturation or residue of myosin, which plays a key role in the quality of meat due to its ability to retain water (37).

**Figure 2:**
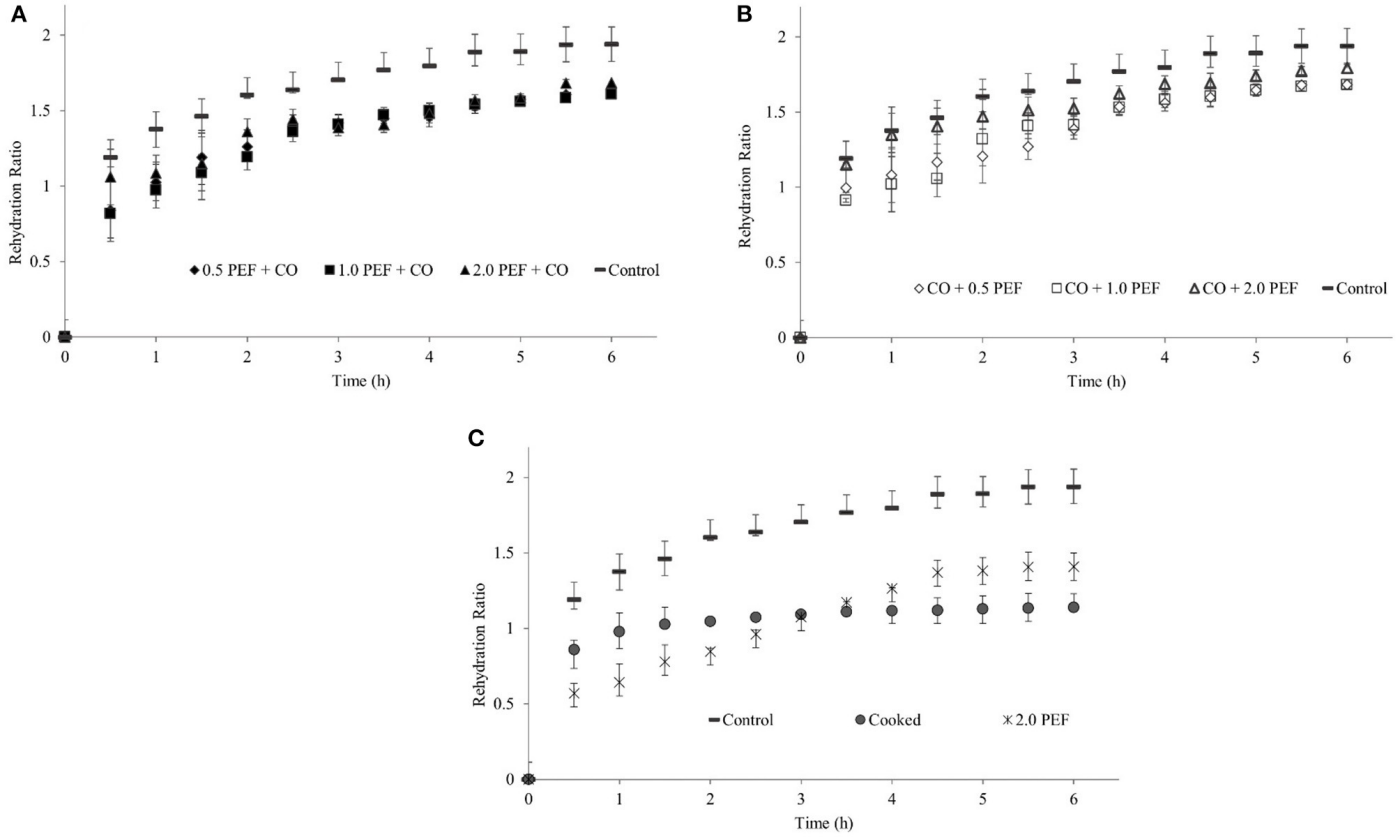
Rehydration kinetics of freeze-dried samples under different pretreatment conditions: **(A)** PEF + CO pretreated samples compared to control sample, **(B)** CO + PEF pretreated samples compared to control sample, and **(C)** 2.0 PE or CO pretreated samples compared to control sample.

Figure 2 shows the characteristic behavior of the rehydration kinetics of pretreated and non-pretreated freeze-dried samples, where the water absorption was faster than the process from the beginning until an equilibrium moisture content is reached. The high water absorption at the beginning of the process can be due to the filling of the superficial capillaries. Then, as the freeze-dried samples continue water absorption, the velocity begins to slow down due to the filling of the intercellular spaces and capillaries. Typically, FD techniques showed higher rehydration rates compared to other techniques, such as VD, but required a longer process (38).

Although there were no statistically significant differences when comparing to pretreated samples and control (Figure 2), the results obtained are similar to those reported by Aksoy et al. (38) for freeze-dried minced meat. They presented rehydration ratios of 1.27, 1.31, and 1.93 for VD, ultrasonic vacuum (USV), and FD, respectively. This similarity might be due to PEF pretreatment, which could cause an effect like meat grinding, this trend is also reported by Aravindakshan et al. (40), who compared rehydration kinetics for the different drying methods: HD, VD, and FD.

For protein content, the results trend indicated that protein content is closely related to moisture content. Table 1 shows statistically significant differences in the samples pretreated with CO + 2.0 PEF and 2.0 PEF + CO have a higher protein content of 84.3% and 83.7%, respectively, along with the lowest moisture content. In addition, it is worth mentioning that the protein values presented in this research were comparable to those reported by Reyes et al. (3) and Briones-Labarca et al. (23), who worked with MW and FD in Chilean abalone, and with high hydrostatic pressure in red abalone, respectively. Differences in protein values were associated with the intrinsic characteristics of each mollusk as well as heat and pressure treatments.

### Texture Profile Analysis

Texture profile analysis results for PEF and CO pretreated freeze-dried samples are shown in Table 2. According to Ling et al. (41), the textural parameters, such as hardness, elasticity, and firmness, were measured to evaluate thermal degradation thereof. While for Chilean abalone, the cohesiveness and chewiness parameters must also be considered (23) because drying processes denatured protein, which induced irreversible structural changes, i.e., texture (42, 43).

**Table 2:** Texture profile analysis (TPA) results for freeze-dried Chilean abalone samples pre-treated by combined pulsed electric fields (PEF) and cooking (CO) conditions.

**Treatments**	**Hardness (N)**	**Springiness (cm)**	**Cohesiveness**	**Chewiness (cm)**
Control	473 ± 17^a^	0.9 ± 0.1^c^	0.8 ± 0.1^b^	33.0 ± 4.5^a^
CO+0.5 PEF	206 ± 11^d^	1.0 ± 0.1^ab^	0.8 ± 0.0^ab^	16.9 ± 1.5^de^
CO+1.0 PEF	304 ± 24^b^	0.9 ± 0.0^b^	0.8 ± 0.0^ab^	24.6 ± 1.5^bc^
CO+2.0 PEF	311 ± 25^b^	1.0 ± 0.1^a^	0.9 ± 0.0^a^	28.4 ± 4.6^ab^
0.5 PEF+CO	155 ± 14^e^	1.0 ± 0.0^ab^	0.8 ± 0.0^ab^	12.6 ± 1.2^e^
1.0 PEF+CO	251 ± 33^c^	0.9 ± 0.0^b^	0.9 ± 0.0^ab^	20.7 ± 3.7^cd^
2.0 PEF+CO	244 ± 22^c^	1.0 ± 0.0^ab^	0.8 ± 0.1^ab^	19.8 ± 1.9^cd^
Cooked	216 ± 15^d^	1.0 ± 0.1^ab^	0.8 ± 0.0^ab^	18.9 ± 1.4^de^
2.0 PEF	213 ± 31^c^	0.9 ± 0.1^a^	0.9 ± 0.1^ab^	17.8 ± 5.0^de^

*Lowercase letters indicate the statistical significance (p < 0.05) between all the treatments compared with each other (column)*.

From Table 2, the results showed the highest hardness values (473 N) for the control sample, whilst other treated samples presented lower values from 155 to 310 N. However, 1.0 and 2.0 PEF pretreated samples did not present significant differences, whether the CO pretreatment had been applied before or after applying PEF. Regarding 0.5 PEF treated samples, lowest hardness values, however, the sample treated with PEF + CO had the lowest value, 155 N. As to 0.5 PEF treated samples, these showed the lowest hardness values, with values of 155 and 206 N for the treatments 0.5 PEF + CO and CO + 0.5 PEF, respectively. Briones et al. (23) in a study on red abalone evaluated elasticity, cohesiveness, and chewiness parameters for fresh samples when they found the values similar to control samples in this study. However, Briones (23) concluded that the hardness parameter between Chilean abalone and red abalone might be different, mainly due to the feeding ingredients, the first being a carnivore animal and the second being a herbivore.

Chewability parameter presented the results with a similar tendency to hardness, that is, the highest value was 33.0 N for the control sample and the lowest value of 12.6 N for the 0.5 PEF + CO pretreatment. Pankyamma et al. (44) compared the hardness, elasticity, and chewiness parameters for solar drying, HD, and vacuum MW drying in squid, and they reported decreasing texture parameter values after rehydration. Reyes et al. (3) stated that the FD process generates changes in connective tissues and myofibrillar proteins that cause the hardening of the Chilean abalone after rehydration. These authors freeze-dried Chilean abalone under vacuum and atmospheric pressure conditions, along with the kind of sample geometry (laminar and cube), where, in most cases, the hardness increased after rehydration. Thus, this research verified the effect of PEF and CO pretreatments on the textural properties, mainly, in terms of hardness and chewiness (Table 3).

**Table 3:** Enthalpy, fusion temperature, specific heat, and glass transition temperature corresponding to differential scanning calorimetry (DSC) thermograms of pretreated freeze-dried Chilean abalone samples.

**Treatments**	**ΔH (J g^**−1**^)**	**Tm (**°**C)**	**ΔCp (Jg k^**−1**^)**	**Tg (**°**C)**
Control	2.24 ± 0.08^a^	181.52 ± 7.21^a^	0.281 ± 0.10^b^	104.0 ± 0.2^a^
CO+0.5 PEF	1.52 ± 0.23^ab^	176.27 ± 2.05^a^	0.131 ± 0.02^b^	106.0 ± 3.7^a^
CO+1.0 PEF	1.30 ± 0.52^ab^	166.13 ± 7.44^a^	0.040 ± 0.03^b^	104.4 ± 4.2^a^
CO+2.0 PEF	1.37 ± 0.25^ab^	165.08 ± 9.58^a^	0.010 ± 0.00^b^	106.9 ± 0.0^a^
0.5 PEF+CO	1.38 ± 0.05^ab^	167.30 ± 0.89^a^	0.053 ± 0.03^b^	105.3 ± 4.0^a^
1.0 PEF+CO	1.30 ± 0.27^ab^	168.33 ± 8.46^a^	0.099 ± 0.03^b^	106.8 ± 0.0^a^
2.0 PEF+CO	1.08 ± 0.20^b^	160.63 ± 3.20^a^	0.147 ± 0.07^b^	114.6 ± 3.3^a^
Cooked	1.34 ± 0.25^ab^	166.50 ± 7.40^a^	1.190 ± 0.32^a^	106.7 ± 0.7^a^
2.0 PEF	1.36 ± 0.15^ab^	87.08 ± 4.80^b^	0.070 ± 0.01^b^	67.3 ± 0.6^b^

*Lowercase letters indicate the statistical significance (p < 0.05) between all the treatments compared with each other (column)*.

Thermal processing modifies the sensory and textural properties of seafood products partly due to the denaturation of proteins (37), which has been already confirmed by the results of thermal analysis reported in this work. Øiseth et al. (45) studied the texture of abalone and attribute these changes to the fact that muscular cells are mainly fibrils, sarcoplasm, and connective tissue, particularly collagen.

### Thermal Behavior of Proteins

No statistically significant differences were observed in the evaluation of Tg and Tm, but in the ΔH evaluation, the most prominent difference was observed between the 2.0 PEF + CO pretreatment and control sample. The ΔH value correlates with the net content of the ordered secondary structure of a protein, so this factor will be discussed later in the FTIR analysis (46). Conversion from a native protein to heat-denatured is a cooperative phenomenon and is accompanied by significant heat absorption (46). However, the results obtained indicate that the obtained curves may be a result of aggregation and not denaturation. The positive contribution of ΔH is associated with the disruption of hydrogen bonds, which depends on temperature (47), is higher with the decrease in temperature peak (Table 2), and is also associated with the breaking of hydrophobic bonds (47). From a chemical point of view, disulfide bonds and free thiol groups would be responsible for thermal aggregation (48). Then, as there was no aqueous medium that allowed the cleavage of sample proteins, Tm and Tg presented higher values than raw materials (49).

ΔH values for actin and myosin were in the range between 50–70 and 74–84°C, respectively (16). Reyes et al. (5) reported that Cp gradually decreased at a higher CO temperature of Chilean abalone, and also indicated that this is not the case for shellfish, e.g., shrimp or oysters, whose difference could be due to different proximal components. Otherwise, the comparisons made at Tm for the same magnitude of PEF (data not shown) showed that there were statistically significant differences between 0.5 PEF pretreatments as well as 2 PEF for Cp. These results were consistent with the works performed under PEF pretreatments (47, 48, 50).

As for the DSC analysis, direct evidence of Tg was characterized by an increase in ΔCp of the treated samples (51), i.e., when Cp increased, Tg of the sample can be determined by the midpoint in the Cp change. However, Roos et al. (52) suggested that higher moisture content represents a lower Tg value, which can be verified in the results presented in Tables 1, 2.

### Protein Conformational Changes

Table 4 shows the second-derivative FTIR spectra of the Amide I region (1,600–1,700 cm^−1^) obtained from non-pretreated (control) and pretreated freeze-dried samples. These results identified diverse conformations of the protein secondary structure under similar wavelengths, specifically for an intermolecular β-sheet, an intramolecular β-sheet, and β-turn structures, which were also reported by Cepero-Betancourt et al. (53); Larrea-Wachtendorff et al. (16), Mobili et al. (54), and Tabilo-Munizaga et al. (55), both control and treated samples. However, there was a significant difference between an intramolecular structure and a random coil structure for 2.0 PEF + CO pretreatment. These differences could be explained by the presence of lipids and water molecules in small quantities (56), which would induce changes in the intensity of the curve.

**Table 4:** Wavelength (cm^−1^) assignment of protein secondary structures in Amide I region of pretreated freeze-dried samples determined by Fourier transform IR (FTIR) spectroscopy.

**Protein secondary structure**	**Control**	**CO+0.5 PEF**	**CO+1.0 PEF**	**CO+2.0 PEF**	**0.5 PEF+CO**	**1.0 PEF+CO**	**2.0 PEF+CO**
Intramolecular β-sheet	1,612–1,613	1,620–1,623	1,623	1,623	1,621–1,623	1,621–1,622	1,621
β-coil	1,630–1,631	1,634–1,636	1,636	1,636–1,638	1,636–1,638	1,636	1,631–1,638
α- helix	1,646–1,648	1,650–1,652	1,652	1,650	1,651–1,652	1,650	1,650–1,652
Intermolecular β-sheet	1,670–1,672	1,668–1,670	1,668–1,671	1,669	1,668–1,671	1,666–1,669	1,669
β-turn	1,680–1,681	1,682–1,685	1,684	1,682–1,684	1,684	1,682–1,684	1,685

**Figure 3:**
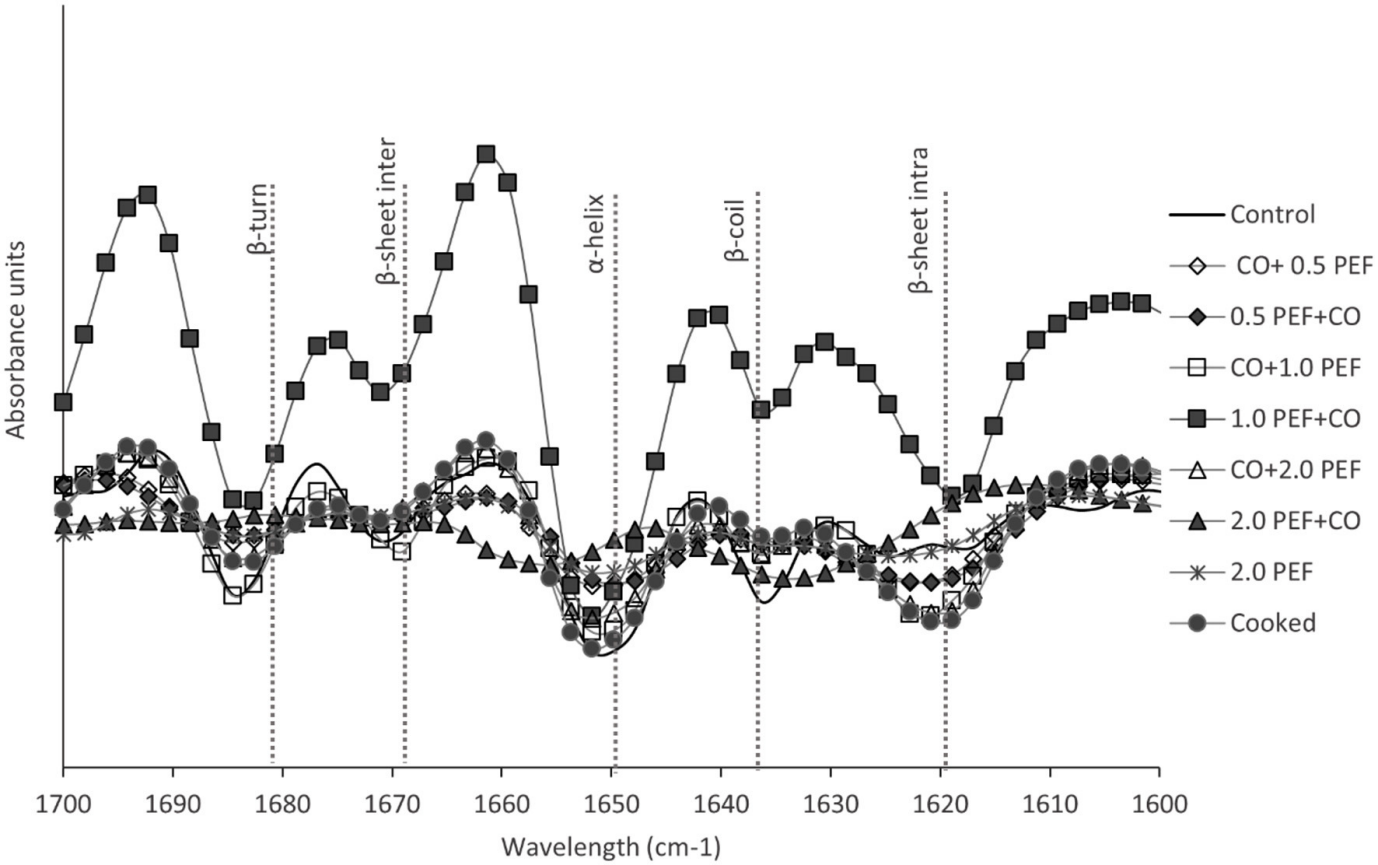
Second-derivative Fourier transform IR (FTIR) of freeze-dried samples under different pretreatment conditions, including the control sample.

On the other hand, Figure 3 shows the second-derivative FTIR spectra of the Amide I region (1,600–1,700 cm^−1^) obtained from CO + PEF pretreated freeze-dried samples as well as the control sample. A comparison between the control sample and freeze-dried samples pretreated with 0.5 PEF + CO and CO + 0.5 PEF showed β-sheet regions, intramolecular at 1,620 cm^−1^, and to a lesser degree, the intermolecular structure at 1,630 cm^−1^. Lu et al. (57) also related he high degree of protein aggregation or intermolecular interactions showed FTIR analysis for freeze-dried protein-excipient mixtures. Embaby et al. (58) and Vasconcelos et al. (59) reported that the accurate positions and shapes of the peaks were due to other compounds of samples either on their own or in addition. In general, the most accentuated structure is α-helix, which is due to the CO pretreatment at 100°C. First, Dong et al. (60) informed a correlation coefficient of value 0.99 for this structure with temperature, and then Larrea-Wachtendorff et al. (16) reported the association of a decrease in the α-helix structure with proteolytic degradation.

### *In vitro* Gastrointestinal Digestibility

**Figure 4:**
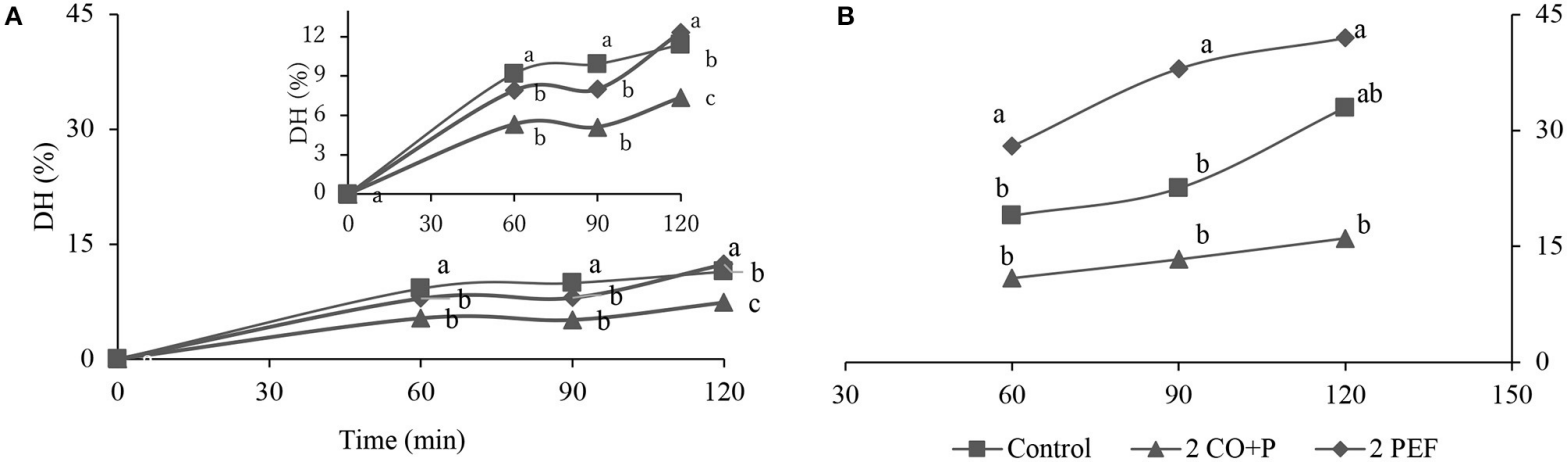
Degree of hydrolysis (DH) of the digested samples, **(A)** the gastric phase at 0, 60, 90, and 120 min and **(B)** the duodenal phase at 60, 90, and 120 min. Lowercase letters indicate statistically significant (*p* < 0.05) differences.

Figure 4 shows the results of degrees of hydrolysis of freeze-dried Chilean abalone samples pretreated under the two conditions: CO + 2.0 PEF and 2.0 PEF. The selection of these pretreatments was due to the shortest FD times, which is shown as FD-kinetics curves (Figure 1). Also, the FD-kinetics of the control sample was used to compare thereof.

In the gastric phase, there were significant differences (*p* < 0.05) for the digestion times between 60 and 90 min, for both cases, the control sample presented the highest DH values. Once this stage was taken, all the selected samples showed statistical differences. As for the duodenal phase, the statistical analysis shows that DH obtained at the end of the digestion was only significantly different (*p* < 0.05) for the samples with 2.0 PEF and CO + 2.0 PEF pretreatments, while it is opposite in comparison to the control sample. The difference between samples 2.0 PEF (higher DH value) and CO + 2.0 PEF (lower DH value) was around 33.4%. DH differences between the selected pretreatments could be related to the protein conformational changes induced by processing (53). Furthermore, Li et al. (61) reported slight differences between cooked meat products, which were attributed due to endogenous enzymatic proteolysis and the possible extraction or loss of soluble proteins (myofibrillar) during CO processing. According to Pak et al. (62), they reported apparent digestibility for Chilean abalone, a value of 83.6%, which is the lowest value when compared with other shellfish such as clam (90.4%). Chibor and Kiin (63), who presented the highest digestibility values for oysters of around 16.75%, demonstrated that the digestibility values for seafood products decrease drastically when they are evaluated fresh *in vitro*.

However, Li et al. (61) determined DH for combined CO pretreatments in clams, and the same trend presented in this article was obtained, meaning that the highest DH values were for the pretreated sample with a non-combined condition, followed by the control sample and combined pretreatments. It has been reported that protein unfolding could expose the sites prone to the action of proteases in matrices of plant origin, but in this case, there are endogenous proteases involved in the enzymatic hydrolysis from native muscle proteins (61).

To define protein quality, the amino acid composition is the most important factor, followed by digestibility (7, 28). Table 5 shows the Computed Protein Efficiency Rate (C-PER) values for the digested Chilean abalone samples under the selected conditions: 2.0 PEF and CO + 2.0 PEF. CO + 2.0 PEF pretreatment was the only one showing significant differences between 0 and 90 min in the gastric phase, while the duodenal phase showed significant differences for pretreatment 2.0 PEF in all-time measurement. Phimphilai et al. (26) reported C-PER values for three different matrices, two vegetal and one animal origin, and no differences were found between them. However, differences between vegetal origin samples could be attributable to pH values from the industrial processing and also a possible buffer capacity in each sample. Oduro et al. (64) evaluated C-PER in chub-mackerel and reported the highest values for the cooked samples, which may be ascribed to the protein bioavailability changes in each process.

**Table 5:** C-PER for *in vitro* digested samples under gastric and duodenal phases and times.

**Phase**	**Time (min)**	**Control**	**2 CO + PEF**	**2 PEF**
Gastric	0	0.09 ± 0.00^a^	0.03 ± 0.00^b^	0.09 ± 0.00^a^
	60	1.04 ± 0.02^a^	0.84 ± 0.02^a^	1.10 ± 0.00^a^
	90	1.12 ± 0.00^a^	0.81 ± 0.05^b^	1.11 ± 0.00^a^
	120	1.21 ± 0.01^a^	1.10 ± 0.23^a^	1.47 ± 0.15^a^
Duodenal	0	–	–	–
	60	1.37 ± 0.05^b^	1.12 ± 0.03^b^	2.87 ± 0.24^a^
	90	1.41 ± 0.14^b^	1.21 ± 0.09^b^	3.81 ± 0.09^a^
	120	0.97 ± 0.04^b^	1.27 ± 0.01^b^	4.18 ± 0.26^a^

*Lowercase letters in each horizontal line indicate statistically significant (p < 0.05) differences*.

### Microstructural Analysis

**Figure 5:**
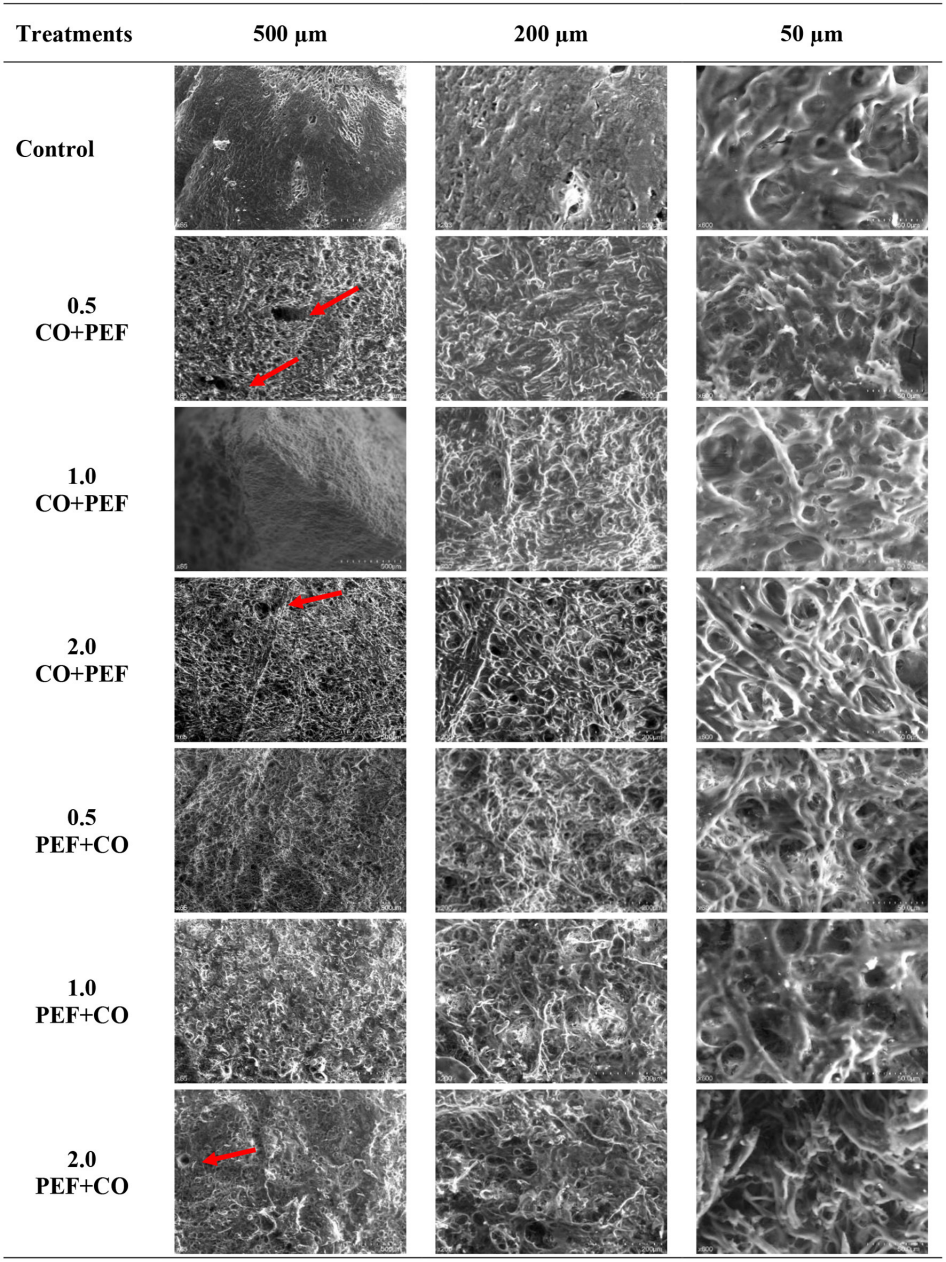
SEM images of pretreated (PEF + CO or CO + PEF) freeze-dried Chilean abalone samples. Red arrows show PEF porosity.

The microstructure images of CO + PEF pretreated freeze-dried samples are shown in Figure 5. Both the control and pretreated samples have porous and amorphous structures like a sponge. The sample's microstructure was compared to abalone treated by high hydrostatic pressure (23), cooked abalone (65), beef muscle treated with PEF (66), and freeze-dried Chilean abalone (3). Based on the foregoing, it can be noticed that the Chilean abalone samples treated by the FD process were the most affected ones, above the CO and PEF pretreatments. However, when comparing the micrographs taken at 50 μm of samples treated by PEF + CO, it was appreciated as a less rigid and more disordered structure than when applying the pretreatments in reverse order. Moreover, the porosity generated by the PEF pretreatment was only noticeable in the micrographs 500 μm, particularly for CO + 0.5 PEF, CO + 2.0 PEF, and 2.0 PEF + CO pretreatments, whose pores were marked with red arrows. Alahakoon et al. (66) reported a more porous surface by applying 1.5 kV cm^−1^. Briones-Labarca et al. (23) working with high hydrostatic pressures applied to abalone muscle, observed a porous structure, whose holes were compressed by high-pressure application. Xin et al. (65), on the other hand, took SEM photographs of abalone muscles cooked at 100°C for 1, 2, and 3 h, and they found different CO times significantly affected the microstructure. Regarding the research carried out by Reyes et al. (3), the Chilean abalone muscle was subjected to FD, and SEM micrographs reported were very similar to those obtained in this research, particularly for images taken at 50 μm.

## Conclusions

Different PEF and CO conditions as pretreatments to Chilean abalone FD have been confirmed to affect processing time and product protein quality. As for the PEF- and CO-assisted FD process, this was reduced considerably by 50% with respect to processes without pretreatments. The FTIR spectroscopy analysis established that the protein structural bonds of Chilean abalone were significantly modified using PEF and CO pretreatments. More specifically, these pretreatments could have affected the hydrogen bonds or the charges of the amino acids, thus producing displacements and variations in the structure intensity. In addition, the DSC analysis showed that CO pretreatment completely denatured the Chilean abalone proteins. However, this denaturalization gave a more rigid structure, so further electroporation was more effective in getting a shorter FD time for this group of pretreatments. As for the microstructure, the microimages showed that the main effect on sample structure was observed due to a kind of pretreatment, i.e., combined PEF + CO or both independently. On the other hand, the order of pretreatments also had a significant effect on final moisture content, WHC, and texture parameters of freeze-dried Chilean abalone samples. With respect to this, the 2.0 kV cm^−1^ PEF + CO pretreatment presented the most significant quality changes in Chilean abalone samples, which showed that the pretreatment order is an important factor to consider for future study or research. As for the digestibility study, both the DH and C-PER, they also evidenced that samples treated by 2.0 kV cm^−1^ PEF presented higher values in both analyses. Finally, it can be concluded that CO pretreatment is an important process due to protein quality can be significantly improved with its use. Thereby, the textural parameters being a critical point of the Chilean abalone quality could be strengthened when combining PEF and CO.

## Data Availability Statement

The original contributions presented in the study are included in the article/supplementary material, further inquiries can be directed to the corresponding author/s.

## Author Contributions

RL-M designed the study, analyzed data, and revised the manuscript. JO-V conducted the experiments and wrote the manuscript draft. AP-A conducted the experiments and wrote part of the manuscript draft. MP-W conducted the experiments and analyzed data. GT-M participated in analyzing results and revising the manuscript. All authors contributed to the article and approved the submitted version.

## Conflict of Interest

The authors declare that the research was conducted in the absence of any commercial or financial relationships that could be construed as a potential conflict of interest.

## Publisher's Note

All claims expressed in this article are solely those of the authors and do not necessarily represent those of their affiliated organizations, or those of the publisher, the editors and the reviewers. Any product that may be evaluated in this article, or claim that may be made by its manufacturer, is not guaranteed or endorsed by the publisher.
